# Model of Love, Hate, and Anxiety Scripts in Psychopathic Individuals

**DOI:** 10.3389/fpsyg.2015.01722

**Published:** 2015-11-13

**Authors:** Barbara Gawda

**Affiliations:** Department of Psychology of Emotion and Cognition, Maria Curie-Skłodowska-UniversityLublin, Poland

**Keywords:** narrative analysis, emotional scripts, psychopathy, love, hate, anxiety

The narrative studies provides considerable information related to the affective sphere in individuals with antisocial and/or psychopathic personality. Narrative studies on emotions of psychopaths revisits past efforts to establish a “linguistic typology” for antisocial personality (Rieber and Vetter, 1994).

The studies on emotional narratives have showed ambivalence of the scripts of love, hate, and anxiety in psychopathic individuals. In the script of love the actor is shown as both positive and negative, experiencing joy and happiness as well as sadness, anger, and aversion. The description of a partner also lacks clarity in terms of the positive/negative emotions. The valence of the script of hate is also unclear. The character traits of the actor are extremely positive while those of the partner are negative, just like their emotions. The picture of hatred contains numerous inadequate “toward-type” actions as well as positively perceived endings of the scene. Similarly to love and hate, the script of anxiety is also of ambivalent nature. The findings suggest the ambivalent nature of the scripts of complex emotions in psychopaths. This means their emotional sensations are vague and unclear; these individuals cannot accurately identify the correct meaning of various affective situations (Gawda, 2010). The ambivalence may lead to inaccurate identification of goals, aspirations, and intentions.

The findings correspond with a relatively new approach to the affective functioning of psychopaths according to which those individuals experience emotions without clearly recognizing their valence, i.e., in ambivalent manner. This is consistent with reports explaining the mechanism of ambivalence taking into account the analysis of ineffectiveness of mutual suppression of BAS and BIS systems (Newman et al., 2005).

In accordance with the concept of script structure, the representation of a given emotion should contain information related to conditions preceding a given affective scenario (explanation of causes), assessment of people (oneself and others) involved in an affective scenario, actions typical for a given emotional scene and ways of ending it. In the case of the scripts of love, individuals with psychopathic personality were able to identify the causes by pointing to positive events preceding the emotion of love. While depicting themselves as the main actor of the scene, they pointed to positive emotions, yet there were also negative descriptions. The comparison of the number of positive descriptions of oneself and the partner shows that individuals with psychopathy far more often use positive terms to describe themselves than the partner (Gawda, 2013). Furthermore, they do so with a great conviction. This is a manifestation of their increased focus on themselves, and an expression of characteristics of psychopathic personality, such as the sense of grandeur and bloated self-esteem. Even though they accurately identify the causes of events and adequately recognize typical related actions, individuals with antisocial personality cannot clearly estimate the ending for the event. This may be linked with the ambivalent approach to love. Attributing love with negative elements contributes to the difficulties in assessing the possibility to realize a given emotional scenario.

Representation of hate should contains particularly negative image of the object of hate. Yet it was demonstrated that the scripts of hate in psychopaths are not so explicitly negative. These individuals perceive their personal characteristics as extremely positive. They believe they are good, agreeable, and capable of solving problems. The partner in a scene of hate is far worse in terms of the experienced emotions and qualities of character. Psychopathic individuals cannot identify the causes of an event leading to the emotions of aversion, hostility, anger, and hate. It is also difficult for them to describe actions which are typical of hatred and their predictions regarding the conclusion of the scene are equally inadequate. Their opinions concerning hatred, formulated in a rather characteristic manner, are emotionally loaded, and generalized (Gawda, 2010). This suggests cognitive rigidity and implies that a number of defensive mechanisms are employed to reduce tension in a situation of hatred, such as denial, underestimation, depreciation of others, and self-idealization (expressed belief regarding one's excellent competences for problem solving). The script of hate structured this way prevents adequate insight and response or conclusions from previous emotional experiences (Figure 1).

**Figure 1:**
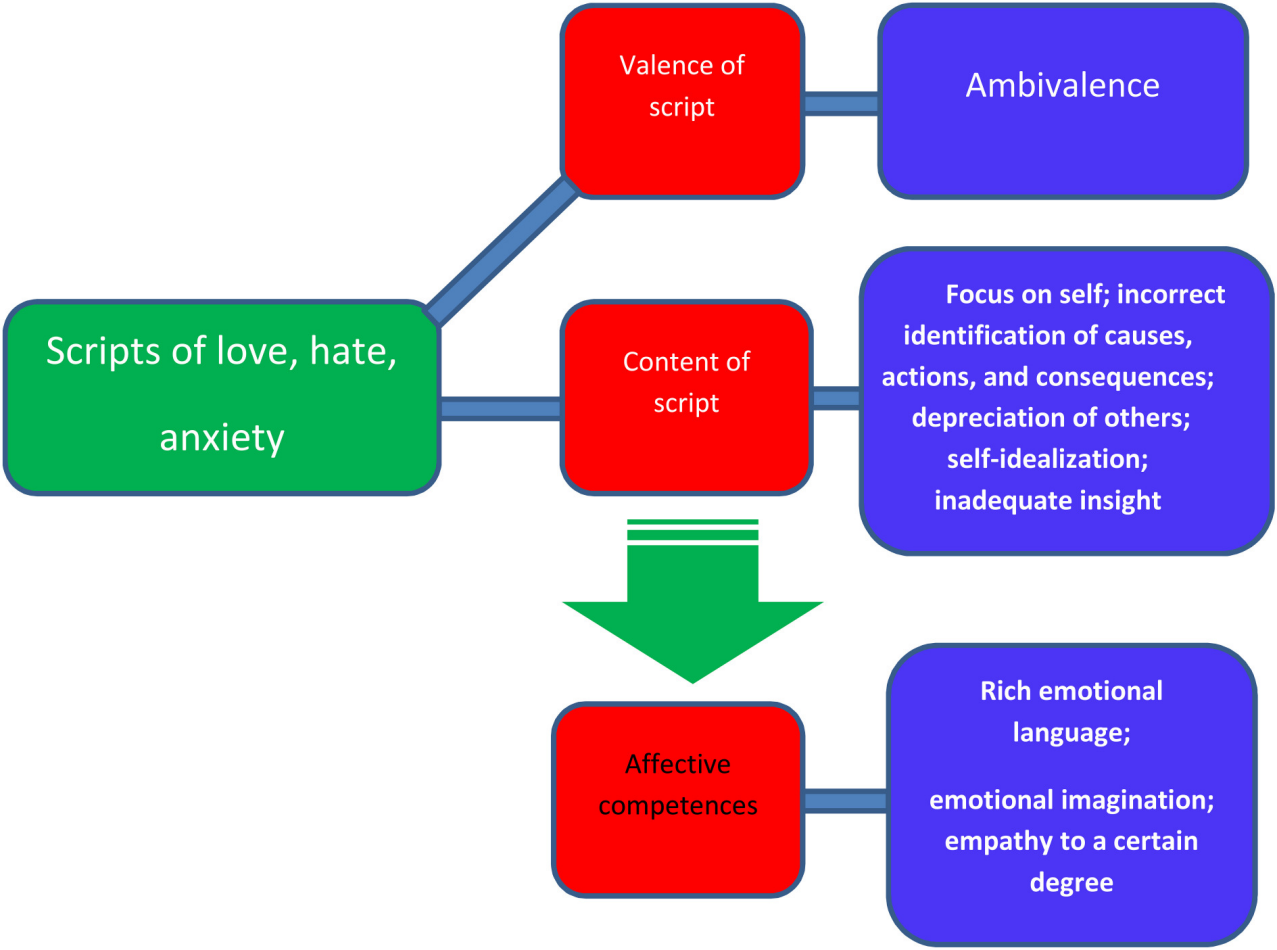
**Model of scripts of complex emotions in psychopaths**.

Anxiety related cognitive pattern in a situation of loneliness should have negative valence. Individuals with psychopathy correctly identify the causes of anxiety yet they cannot clearly describe their emotional state, even though many expressions are chosen correctly. They also exhibit difficulties in characterizing persons involved in a scene of anxiety in terms of their emotions. It is quite difficult for them to correctly determine actions which are typical in a situation of anxiety. Yet the dominant type of action indicated by psychopathic individuals correlates with the way the scene ends. They express their hope that the situation of frustration will end, and deprivation of the basic needs will change into the fulfillment of needs. Mechanisms frequently displayed in this case include idealization, denial, and dogmatic approach to issuing opinions. The above contents of scripts illustrate numerous cognitive impairments in the process of drawing conclusions by people with the antisocial personality disorder (Gawda, 2013). The demonstrated inaccuracy of perceptions related to current situations is manifested in the tendencies to exaggerate (e.g., bloated self-esteem), minimize (e.g., underestimation of partner's emotions), or over-generalize (formulating judgments, categorical statements). Psychopathic individuals in a way screen information in order to highlight their own competences and at the same time reject or ignore critical information.

Analysis of the structure and contents of emotional narratives written by individuals with antisocial personality provided significant insight into some of their affective competences. These individuals have verbal affective competences. This means they can skillfully use emotional language. They have rich emotional vocabulary, are able to describe affective phenomena and use emotional lexicon. This does not mean that they correctly identify the nature of emotional phenomena; yet an assessment of the narrative expressions shows a wealth of structures. Based on the findings regarding the structure of the narratives related to love, hate, and anxiety produced by individuals with psychopathy and antisocial personality it is possible to draw conclusions about their emotional imagination. These individuals are able to imagine an affective scene with all its components. The description of the situation is correct in terms of the structure, but it suggests difficulties in assessing an event for its adequacy and predicting its consequences. The ability to quite precisely describe affective experiences of characters appearing in the story suggests the subjects display elementary empathic capacities. For instance they can understand the situation of an abandoned child. Tremendous emotional involvement accompanying such descriptions confirms empathic traits in the subjects with psychopathy. Yet, these empathic abilities seem to be of a specific kind; in a situation when their sense of self-esteem is threatened (in a situation of hate) these individuals do not try to understand the partner's emotions, and categorically depreciate his/her capacities while emphasizing their own competencies. In a situation of love they are unable to adopt the partner's viewpoint and with unwavering certitude credit themselves with significantly more positive qualities. Hence, individuals with psychopathic personality are able to empathize, but only to a degree, i.e., as long as there is no conflict with their own sense of self-esteem. This means there is a capacity to control empathic behaviors up to a certain level, yet once “self” is threatened defensive mechanisms are automatically started to eliminate the feeling of discomfort.

Hatred is a specific and complex emotion whose script is quite elaborate in psychopathic individuals. It may be assumed that such traits as ruthlessness, tendency to manipulate, narcissism, and excessive self-esteem constitute a mechanism of controlling tension in a situation of hatred. Hence, the script of hate contains a perception of oneself as a being that is unique, one of a kind and endowed with such attributes as goodness and agreeableness. This leads to underestimating the situation, and to inadequate assessment of the consequences of and actions related to the situation. Psychopathic traits constitute a cohesive system of personality and in any real or imagined situation threatening one's sense of greatness, they contribute to one's biased interpretation of the reality. This means that due to its specificity, the complex emotion also impacts the development of its representation. Hate is an emotion linked with specific rules related to manifesting it. Its unhindered and unlimited expression is considered to be inappropriate by the society. The display of hatred is linked with sanctions and consequences, also of legal nature. Indeed, the subjects' situation in life is a reflection of this. Therefore, the emotion is liked with an obligation to apply a system of coping with it.

The narrative findings contradict the generally accepted opinion about a deficit of anxiety in psychopathic individuals (*ibid*.). To the contrary, these individuals have a cognitive representation of loneliness-type anxiety, they are able to imagine such a situation, and can describe it using quite rich affective lexicon. They have the capacity to place themselves in a situation of loneliness and to give an account of such a state. Hence, anxiety as a complex emotion, is known to psychopaths. Research also shows that the population of psychopathic individuals includes persons with varied capacities for experiencing anxiety (Lorenz and Newman, 2002). High-reactive psychopathic individuals are able to recognize greater variety of emotions in interacting partners, while low-reactive psychopathic individuals perceive the consequences of their behaviors as negative.

To conclude, psychopathic individuals have representations of both positive and negative emotions, the latter being more elaborate. The representations contain generalized knowledge of affective phenomena, yet the knowledge is vague, particularly in terms of valence; these individuals face difficulties when they attempt to identify the causes, adequately describe the actions related to a given event or predict the consequences. In addition to the affective knowledge psychopathic individuals have certain emotional competences. These individuals employ a number of mechanisms which make it difficult for them to correctly experience emotions (focus on oneself, overestimation, minimization, denial). Representation of emotions in psychopathic individuals also depends on the type of emotion. Emotions exposing “self” to a highest degree are not welcome and on those occasions the largest number of mechanisms are created in order to eliminate these.

## Conflict of interest statement

The Reviewer Aneta Borkowska declares that, despite being affiliated to the same institution as the author Barbara Gawda, the review process was handled objectively and no conflict of interest exists. The author declares that the research was conducted in the absence of any commercial or financial relationships that could be construed as a potential conflict of interest.
